# Asymmetric Puzzle Spreader Graft: A Systematic Approach for Correction of Crooked Nose Deformity

**DOI:** 10.1007/s00266-026-06001-z

**Published:** 2026-07-22

**Authors:** Erkan Yildiz, Mehmet Yurtseven, Çağlar Günebakan, Selçuk Kuzu, Fatih Çelenk, Abdulkadir Bucak, Orhan Kemal Kahveci

**Affiliations:** Department of Otorhinolaryngology, Afyonkarahisar Health Sciences University, Afyonkarahisar, Turkey

**Keywords:** Rhinoplasty, Crooked nose, Spreader graft, Asymmetric osteotomy, Nasal deviation, C-shaped deformity

## Abstract

**Background:**

Correction of crooked nose deformity remains one of the most challenging aspects of rhinoplasty. C-shaped and reverse C-shaped nasal deviations require asymmetric surgical approaches to achieve optimal aesthetic and functional outcomes. Traditional symmetric spreader graft techniques often fail to address the underlying asymmetric deformity adequately.

**Objectives:**

This study aimed to evaluate the efficacy of a novel asymmetric puzzle spreader graft technique combined with asymmetric osteotomy and osteoplasty in correcting C-shaped and reverse C-shaped nasal deviations.

**Methods:**

Between 2021 and 2023, 50 patients (27 males, 23 females; mean age 35.4 years; range 18–50 years) with axial deviation (28 C-shaped, 22 reverse C-shaped) underwent rhinoplasty using asymmetric osteotomy, osteoplasty, and asymmetric puzzle spreader grafts performed by a single surgeon. On the wider convex side, an extended asymmetric spreader graft reaching the radix was placed, while on the narrower concave side, a smaller spreader graft starting from the K-point was inserted. Rhinion deviation angle (RDA) and nasal tip deviation angle (NDA) were measured using GNU Image Manipulation Program (GIMP, version 2.8.22) on frontal photographs. Mean follow-up was 2 years (range: 1–3 years).

**Results:**

Preoperative mean RDA was 7.28° (range: 3.5–17.46°), which significantly decreased to 3.15° (range: 1.1–4.2°) postoperatively (*P* < 0.05). Preoperative mean NDA was 4.67° (range: 2.5–13.01°), which significantly decreased to 1.23° (range: 0.45–3.21°) postoperatively (*P* < 0.05). The mean preoperative Nasal Obstruction Symptom Evaluation (NOSE) score was 62.57 (SD: 19.44; median: 71), which significantly improved to 21.84 (SD: 16.81; median: 16) at 1-year postoperative follow-up (*P* < 0.001). Statistical analysis using Wilcoxon signed-rank test revealed significant improvement in both RDA (56.7% reduction), NDA (73.7% reduction), and NOSE scores (65.1% reduction). Complications included one wound infection (2%), two minor nasal bleeding episodes (4%), and one patient requiring revision (2%).

**Conclusions:**

The asymmetric puzzle spreader graft technique combined with asymmetric osteotomy provides effective correction of crooked nose deformity with significant improvement in objective nasal deviation measurements and low complication rates. This technique addresses the asymmetric nature of the deformity by tailoring graft size and placement to each side’s specific anatomical requirements.

**Level of Evidence IV:**

This journal requires that authors assign a level of evidence to each article. For a full description of these Evidence-Based Medicine ratings, please refer to the Table of Contents or the online Instructions to Authorswww.springer.com/00266.

## Introduction

Crooked nose deformity represents one of the most complex challenges in aesthetic and functional rhinoplasty [1, 2]. These deviations, commonly classified as C-shaped or reverse C-shaped (S-shaped), result from asymmetric cartilaginous and bony structural abnormalities that produce both aesthetic concerns and functional impairment [3, 4]. The correction of these deformities requires a comprehensive understanding of the underlying pathoanatomy and the application of asymmetric surgical techniques tailored to the specific deformity pattern [5].

Spreader grafts, first described by Sheen in 1984, have become a fundamental technique in rhinoplasty for maintaining middle vault width, supporting the internal nasal valve, and correcting dorsal irregularities [6, 7]. Traditionally, spreader grafts have been placed symmetrically on both sides of the septum. However, in cases of crooked nose deformity, symmetric bilateral spreader grafts often prove inadequate because they fail to address the inherent asymmetry of the underlying structural abnormality [8, 9].

Recent literature emphasizes the importance of asymmetric approaches in correcting crooked nose deformities. Demir et al. demonstrated that asymmetric level osteotomy combined with unilateral spreader graft significantly reduced rhinion deviation angle (RDA) from 6.2° to 2.44° and nasal tip deviation from 5.08° to 2.13° at 6-month follow-up [1]. Similarly, Gökler and Koçak reported successful outcomes using a combination of spreader flap and asymmetric spreader graft in 53 patients with crooked nose deformity [2]. Moosavizadeh et al. found that placing unilateral spreader grafts on the convex side of C-type deviations produced superior aesthetic and functional results compared with concave-side placement [10].

The concept of “puzzle grafts” or separated spreader grafts has been introduced to address complex asymmetric deformities more effectively [11, 12]. The term “puzzle” refers to the complementary, interlocking configuration of the grafts, similar to puzzle pieces that fit together to create a stable, integrated framework. These techniques involve customizing graft geometry and placement to match the specific anatomical requirements of each side of the nose, thereby providing a more tailored correction.

Objective measurement of nasal deviation is crucial for assessing surgical outcomes and comparing different techniques. Several methods have been described, including rhinion deviation angle (RDA), nasal tip deviation angle (NDA), and various photographic and three-dimensional imaging techniques [1, 13, 14]. These objective measurements allow for reproducible assessment of surgical results and facilitate evidence-based evaluation of different surgical approaches.

Despite advances in surgical techniques, the optimal approach for correcting crooked nose deformity remains controversial. The combination of asymmetric osteotomy with asymmetric spreader grafting addresses both the bony and cartilaginous components of the deformity, but the specific technique for graft placement and sizing has not been standardized [15, 16].

In this study, we present our experience with a novel asymmetric puzzle spreader graft technique combined with asymmetric osteotomy and osteoplasty for the correction of C-shaped and reverse C-shaped nasal deviations. We hypothesized that by placing an extended spreader graft on the wider convex side and a shorter graft on the narrower concave side, configured to interlock like puzzle pieces, we could achieve more effective correction of the asymmetric deformity. We evaluated the outcomes using objective measurements of RDA and NDA with a mean follow-up of 2 years to provide evidence-based assessment of this technique.

## Materials and Methods

### Study Design and Patient Selection

This retrospective cohort study was conducted at the Department of Otorhinolaryngology, Afyonkarahisar Health Sciences University, between 2021 and 2023. The study was approved by the Afyonkarahisar Health Sciences University Clinical Research Ethics Committee (Approval No: 2023/13) and was conducted in accordance with the Declaration of Helsinki. Informed consent was obtained from all patients.

A total of 50 patients with crooked nose deformity characterized by axial deviation (C-shaped or reverse C-shaped configuration) who underwent rhinoplasty using the asymmetric puzzle spreader graft technique were included in the study. All patients presented with nasal deviation requiring surgical correction and were treated with a combination of asymmetric osteotomy, osteoplasty, and asymmetric spreader grafts.

*Inclusion criteria*:

- Patients with C-shaped or reverse C-shaped nasal deviation
- Age ≥18 years
- Patients requiring surgical correction of nasal deviation
- Availability of adequate preoperative and postoperative frontal photographs
- Minimum follow-up of 1 year

*Exclusion criteria*:

- Previous rhinoplasty (revision cases)
- Significant nasal trauma requiring additional reconstructive procedures
- Congenital nasal deformities (e.g., cleft lip nasal deformity)
- Inadequate photographic documentation
- Lost to follow-up

### Patient Demographics

The study included 50 patients (27 males [54%], 23 females [46%]) with a mean age of 35.4 years (range: 18-50 years). Among these, 28 patients (56%) had C-shaped nasal deviation and 22 patients (44%) had reverse C-shaped deviation. Mean follow-up period was 2 years (range: 1-3 years).

### Surgical Technique

All surgeries were performed by a single surgeon (E.Y.) using an open rhinoplasty approach under general anesthesia.

#### Asymmetric Osteotomy and Osteoplasty

Following standard open rhinoplasty exposure and assessment of the nasal framework, asymmetric osteotomy and osteoplasty were performed to address the bony component of the deviation. The osteotomy pattern was customized based on the direction and severity of the deviation, with the goal of mobilizing the bony pyramid and allowing repositioning toward the midline.

#### Asymmetric Puzzle Spreader Graft Technique

The key innovation of our technique lies in the asymmetric application of spreader grafts tailored to the specific anatomical characteristics of each side. The term “puzzle” refers to the complementary, interlocking configuration of the grafts that fit together to create a stable, integrated framework:

*Convex (Wider) Side*:

- An extended asymmetric spreader graft was harvested from septal cartilage.
- The graft was placed along the dorsal septum, extending from the radix to the caudal septum.
- The graft provided structural support to maintain the corrected position and prevent collapse of the wider side.
- The graft was carved with a complementary shape to interlock with the contralateral graft.

*Concave (Narrower) Side*:

- A smaller asymmetric spreader graft was placed.
- The graft extended from the K-point (keystone area) caudally.
- This shorter graft provided adequate support without over-correcting the narrower side.
- The graft was shaped to complement and interlock with the extended graft on the convex side.

Both grafts were secured with horizontal mattress sutures using 5-0 polydioxanone (PDS) sutures. The grafts were carved to create a smooth dorsal contour and to interlock like puzzle pieces, creating a stable, integrated framework that resists recurrent deviation (Figs .5-7).

The combination of asymmetric osteotomy and asymmetric spreader graft placement allowed for comprehensive correction of both bony and cartilaginous components of the crooked nose deformity.

### Photographic Documentation and Measurement

Standardized frontal facial photographs were obtained preoperatively and postoperatively using a digital camera with consistent lighting, distance (1.5 meters), and patient positioning. Patients were photographed in natural head position with a neutral facial expression.

### Deviation Angle Measurement

Nasal deviation was quantified using GNU Image Manipulation Program (GIMP, version 2.8.22), a validated digital image analysis software [13, 14]. Two angles were measured on frontal photographs:

*Rhinion Deviation Angle (RDA*):

- It is defined as the angle between the vertical facial midline and a line connecting the glabella to the rhinion.
- The vertical facial midline was established by connecting the glabella to the midpoint of the upper lip philtrum.
- It measures deviation at the level of the nasal bones and upper cartilaginous vault.

*Nasal Tip Deviation Angle (NDA)*:

- It is defined as the angle between the vertical facial midline and a line connecting the glabella to the nasal tip defining point.
- It measures deviation at the level of the lower third of the nose.

All measurements were performed by two independent evaluators (E.Y. and M.Y.) who were blinded to the surgical details. Each measurement was repeated three times, and the mean value was used for analysis to ensure reliability.

### Statistical Analysis

Statistical analysis was performed using IBM SPSS Statistics version 20.0 (IBM Corp., Armonk, NY, USA). Continuous variables were expressed as mean ± standard deviation or median (range) as appropriate. The normality of data distribution was assessed using the Shapiro–Wilk test. Since the values were not normally distributed, a nonparametric test was used.

Preoperative and postoperative RDA and NDA values were compared using Wilcoxon signed-rank test for dependent groups. A P value < 0.05 was considered statistically significant.

## Results

### Patient Demographics

A total of 50 patients with crooked nose deformity were included in this study. The cohort consisted of 27 males (54%) and 23 females (46%) with a mean age of 35.4 years (range: 18-50 years). Among the patients, 28 (56%) presented with C-shaped nasal deviation and 22 (44%) with reverse C-shaped deviation. The mean follow-up period was 2 years (range: 1-3 years).

### Rhinion Deviation Angle (RDA)

Preoperative mean RDA was 7.28° (range: 3.5–17.46°). Following surgical correction with the asymmetric puzzle spreader graft technique, postoperative mean RDA significantly decreased to 3.15° (range: 1.1–4.2°) (P < 0.05, Wilcoxon signed-rank test). This represents a mean improvement of 4.13° or approximately 56.7% reduction in rhinion deviation.

The significant reduction in RDA demonstrates effective correction of the upper and middle nasal vault deviation, addressing the bony and upper cartilaginous components of the crooked nose deformity.

### Nasal Tip Deviation Angle (NDA)

Preoperative mean NDA was 4.67° (range: 2.5–13.01°). Postoperatively, mean NDA significantly decreased to 1.23° (range: 0.45–3.21°) (P < 0.05, Wilcoxon signed-rank test). This represents a mean improvement of 3.44° or approximately 73.7% reduction in nasal tip deviation.

The substantial improvement in NDA indicates that the asymmetric puzzle spreader graft technique effectively addresses deviation throughout the entire nasal length, including the lower third of the nose. The 73.7% improvement in nasal tip deviation is notably higher than many reported outcomes in the literature.

### Functional Outcomes: NOSE Scores

The mean preoperative Nasal Obstruction Symptom Evaluation (NOSE) score was 62.57 (SD: 19.44; median: 71), indicating moderate-to-severe nasal obstruction symptoms. At 1-year postoperative follow-up, the mean NOSE score significantly improved to 21.84 (SD: 16.81; median: 16) (P < 0.001, Wilcoxon signed-rank test). This represents a mean improvement of 40.73 points or approximately 65.1% reduction in NOSE scores, demonstrating substantial improvement in patient-reported functional outcomes.

The significant improvement in NOSE scores confirms that the asymmetric puzzle spreader graft technique not only achieves aesthetic correction but also provides meaningful functional benefit by improving nasal airway patency. The reduction in nasal obstruction symptoms validates the technique’s dual benefit for both form and function.

### Statistical Significance

Statistical analysis using Wilcoxon signed-rank test revealed highly significant differences between preoperative and postoperative values for RDA, NDA (P < 0.05), and NOSE scores (P < 0.001), confirming the efficacy of the asymmetric puzzle spreader graft technique in both objectively measurable and patient-reported outcome terms.

### Complications

The complication rate was low in this series. One patient (2%) developed a minor wound infection that resolved with oral antibiotics. Two patients (4%) experienced minor postoperative nasal bleeding that did not require return to the operating room and was managed conservatively. One patient (2%) required revision surgery due to residual deviation at 18 months postoperatively. No cases of graft displacement, extrusion, or major complications were observed.

### Illustrative Cases

Anatomical illustrations (Figs .1, 2, 3, 4, 5 and 6) demonstrate the structural basis and surgical technique of the asymmetric puzzle spreader graft approach. Representative clinical cases showing preoperative and postoperative results are presented in Figs .7-12:

**Fig. 1:**
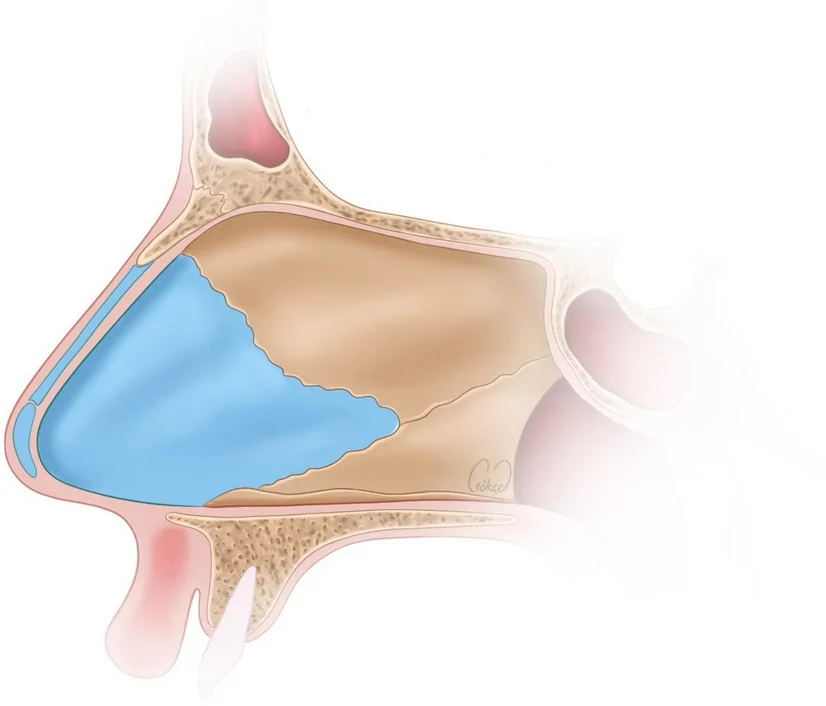
Anatomical illustration showing frontal view of the nasal cartilaginous framework. This illustration demonstrates the underlying structural components and the asymmetry characteristic of crooked nose deformity, providing educational context for understanding the rationale behind the asymmetric surgical approach

**Fig. 2:**
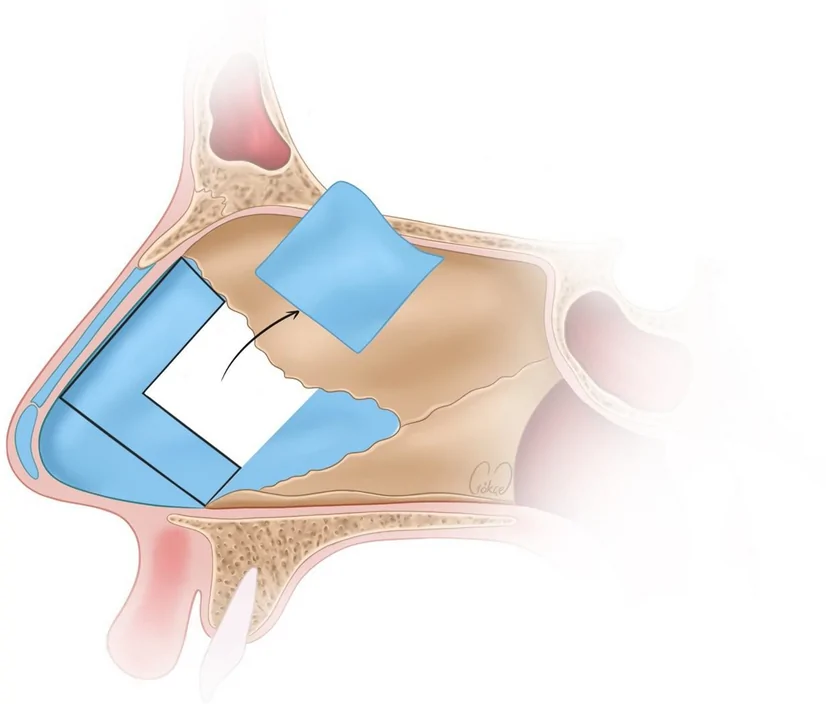
Anatomical illustration showing frontal view with emphasis on the deviation pattern in crooked nose deformity. Note the differential structural abnormalities on the convex versus concave sides, highlighting the biomechanical rationale for asymmetric correction techniques rather than symmetric approaches

**Fig. 3:**
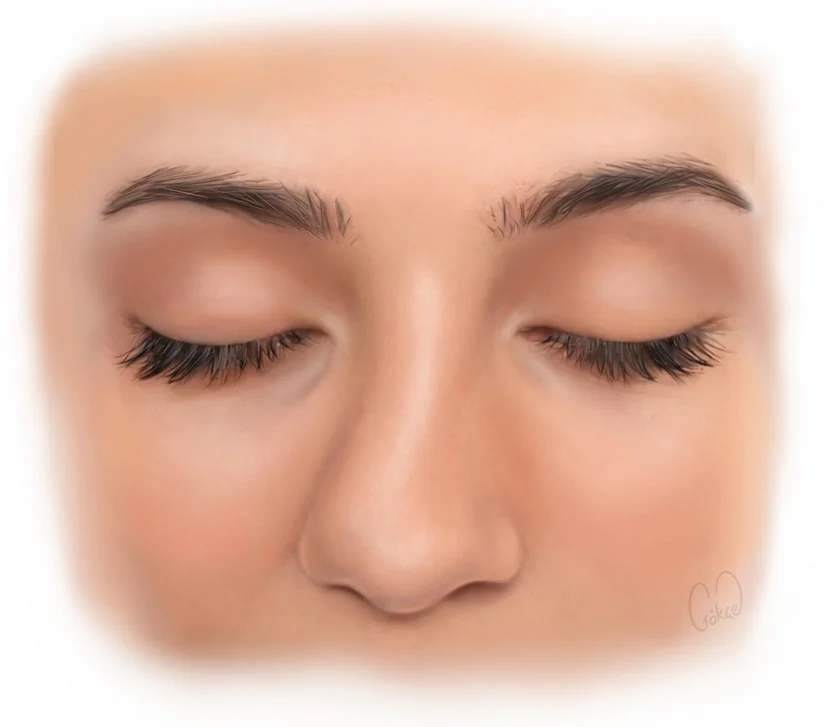
Anatomical illustration demonstrating the corrected nasal position following asymmetric puzzle spreader graft technique. This illustration shows the restoration of midline alignment, improved structural symmetry, and the aesthetic outcome achieved through the asymmetric surgical approach

**Fig. 4:**
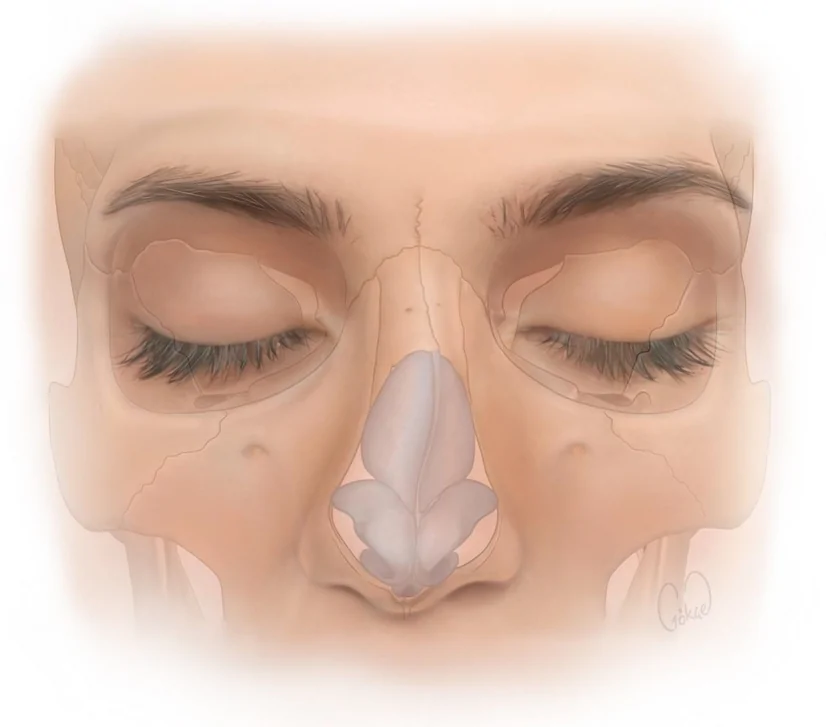
Detailed anatomical illustration showing the nasal cartilaginous framework with anatomical overlay. This illustration emphasizes the three-dimensional nature of the crooked nose deformity and identifies the specific anatomical structures targeted by the asymmetric puzzle spreader graft technique, including the dorsal septum, upper lateral cartilages, and lower lateral cartilages

**Fig. 5:**
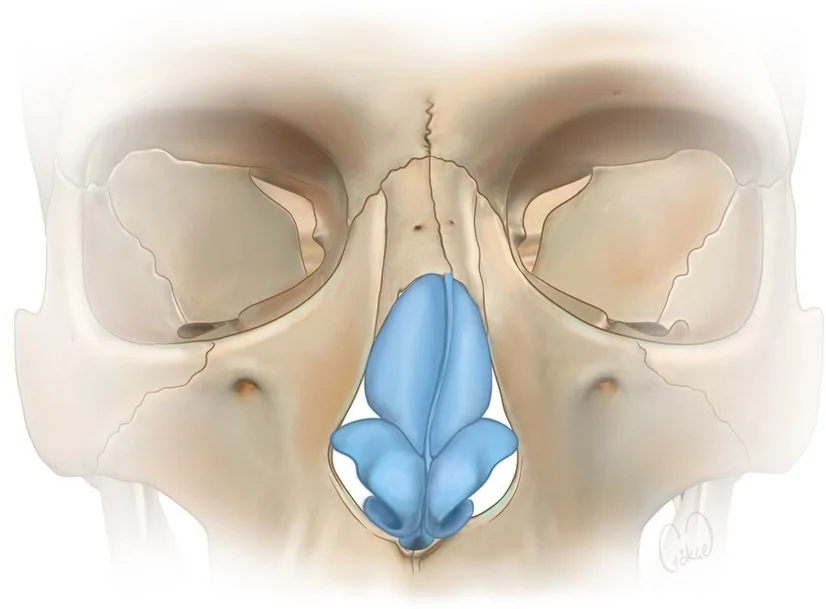
Anatomical illustration showing basal (inferior) view of the nasal cartilage framework, demonstrating the dorsal septum, upper lateral cartilages, and alar cartilages. This basal perspective illustrates how the asymmetric puzzle spreader grafts are positioned relative to the underlying cartilaginous structures and how they provide differential support on the convex versus concave sides

**Fig. 6:**
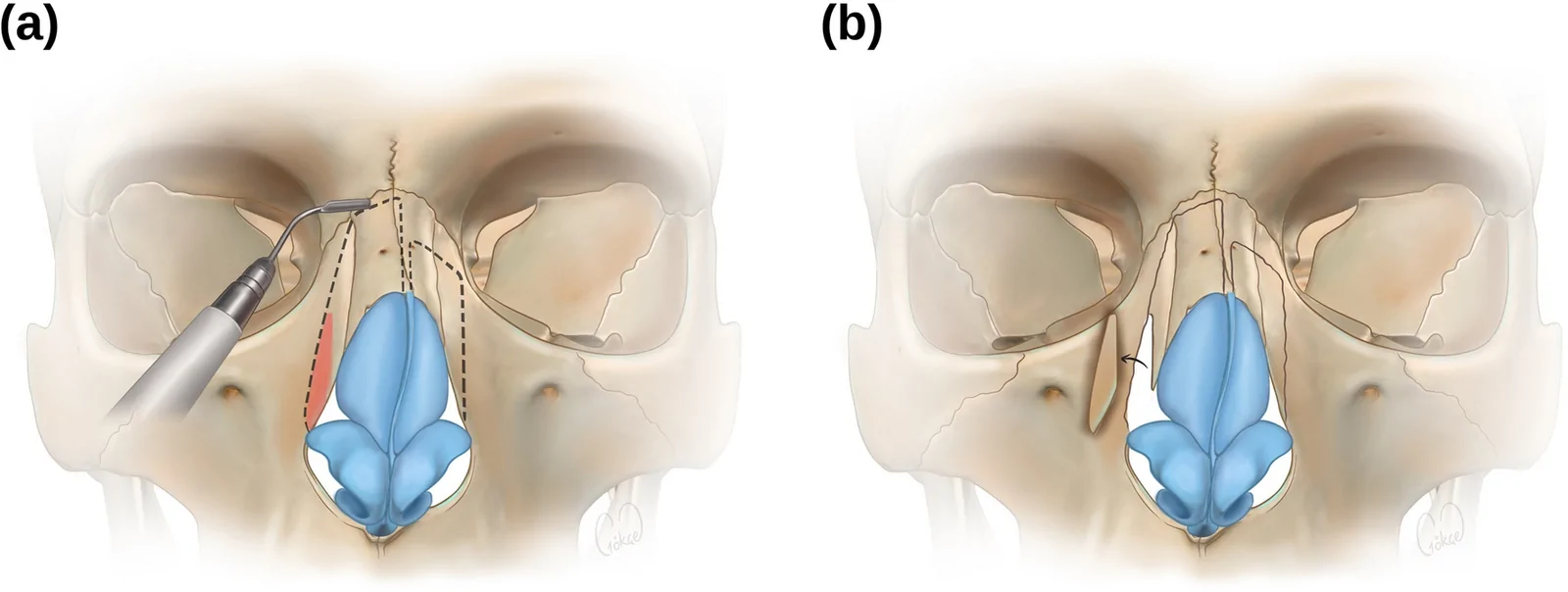
**a** Schematic illustration demonstrating the asymmetric puzzle spreader graft technique in basal view. The extended graft on the wider convex side (green) reaches from the radix to the caudal septum, while the shorter graft on the narrower concave side (red) extends from the K-point caudally. The blue structure represents the nasal tip cartilages. **b** Schematic illustration of ostectomy of the nasal bone, which has a large surface area

**Fig. 7:**
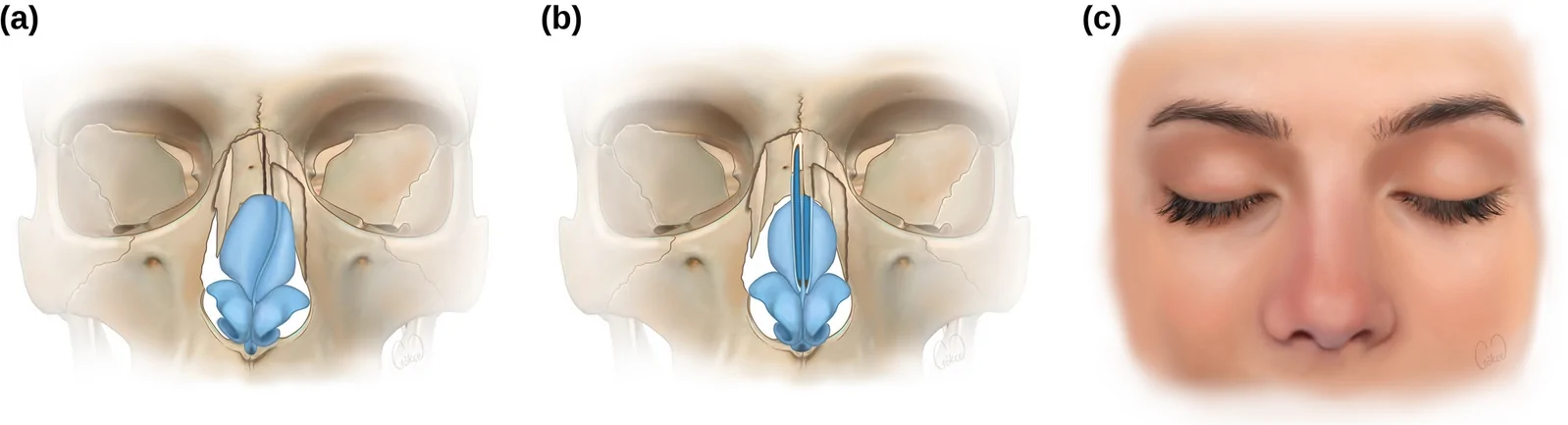
**a** Schematic illustration of the nose after ostectomy. **b** Schematic illustration of the placement of asymmetric puzzle spreader grafts, extended to the radix on the outfracture side and terminated at point K on the infracture site. **c** Schematic illustration of crooked nose correction following placement of asymmetric puzzle spreader grafts

Patient 1 (Fig. 7): Female patient with C-shaped nasal deviation. Left panel shows preoperative frontal view with marked deviation of the nasal dorsum and tip from the facial midline. Right panel shows immediate perioperative view with surgical markings and sutures in place, demonstrating the open rhinoplasty approach and asymmetric osteotomy technique.

**Fig. 8:**
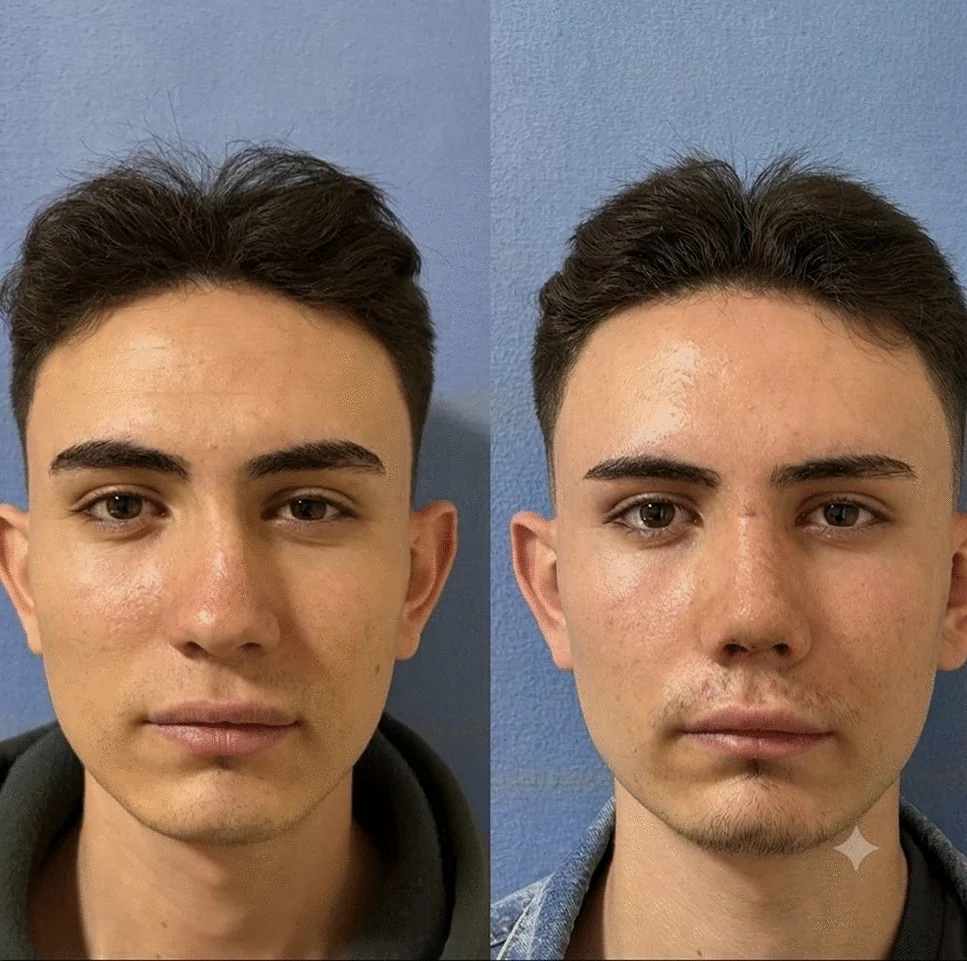
Same patient showing preoperative (left) and postoperative at 2-year follow-up (right). Note significant improvement in nasalaxis alignment, restoration of midlineposition, and improved facial symmetryfollowing asymmetric puzzle spreader graft technique

Patient 1 (Fig. 8): Same female patient showing preoperative (left) and postoperative (right) comparison at 2-year follow-up. Note the significant improvement in nasal axis alignment, restoration of midline position, and improved facial symmetry following asymmetric puzzle spreader graft technique.

**Fig. 9:**
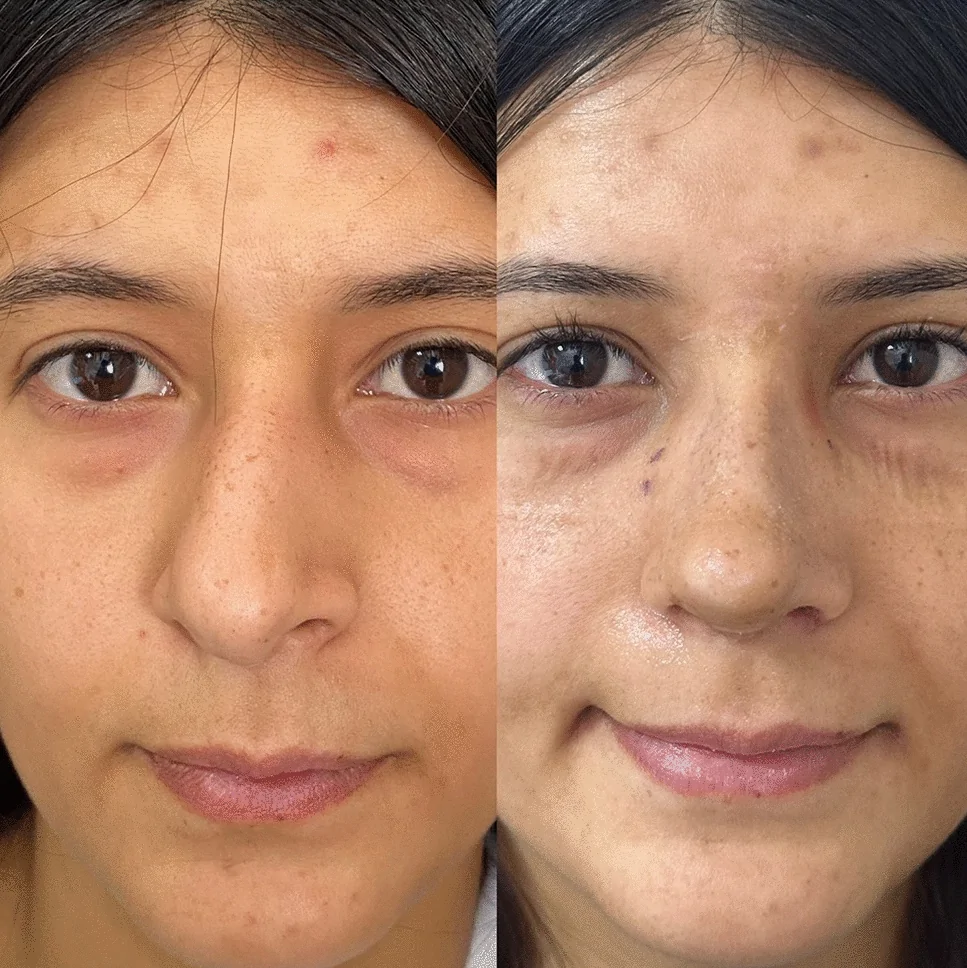
Female patient with C-shaped nasal deviation. Preoperative (left) shows visible dorsal deviation. Postoperative at 18-month follow-up (right) demonstrates excellent correction with straight nasal axis and improved facial harmony

Patient 2 (Fig. 9): Female patient showing preoperative (left) and postoperative (right) frontal views. The preoperative image demonstrates C-shaped nasal deviation with visible dorsal deviation. The postoperative image at 18-month follow-up shows excellent correction with straight nasal axis and improved facial harmony.

**Fig. 10:**
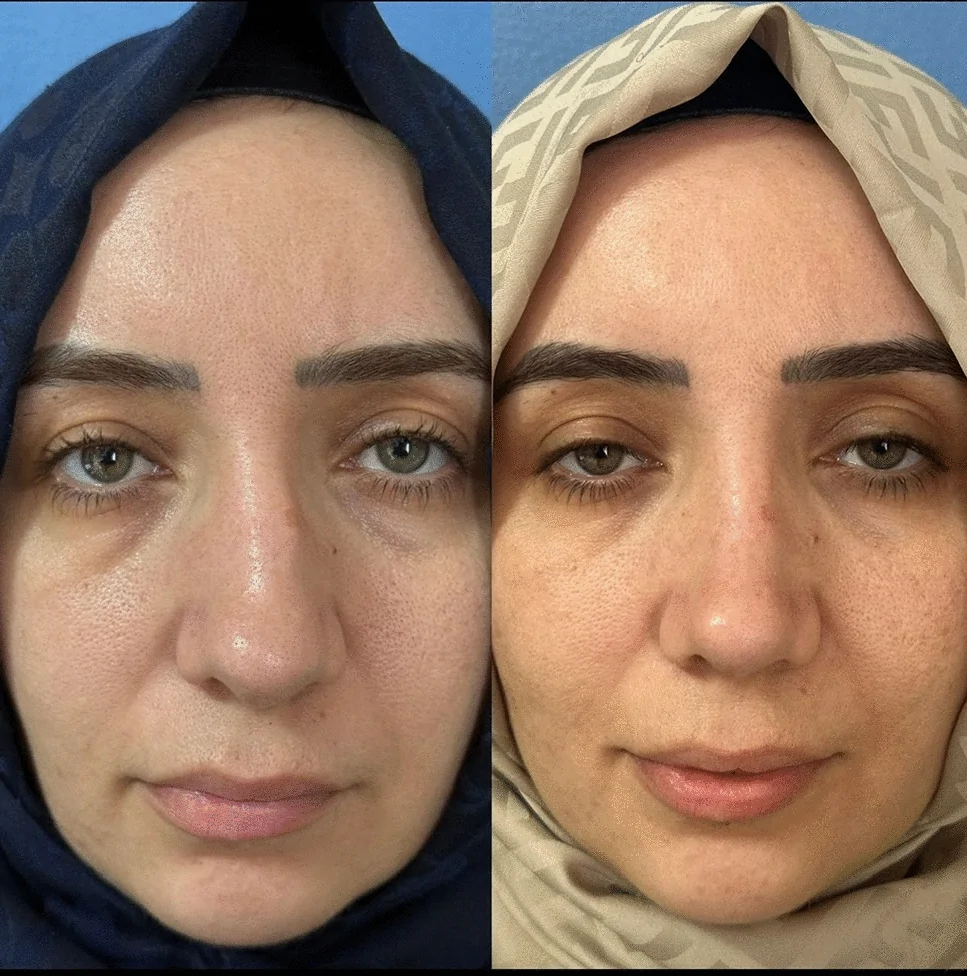
Female patient with reverse C-shaped nasal deviation. Preoperative (left) and postoperative at 2-year follow-up (right) demonstrate successful correction with restoration of nasal symmetry and improved aesthetic outcome

Patient 3 (Fig. 10) Female patient with reverse C-shaped nasal deviation. Preoperative (left) and postoperative (right) comparison demonstrates successful correction of the deviation with restoration of nasal symmetry and improved aesthetic outcome at 2-year follow-up.

**Fig. 11:**
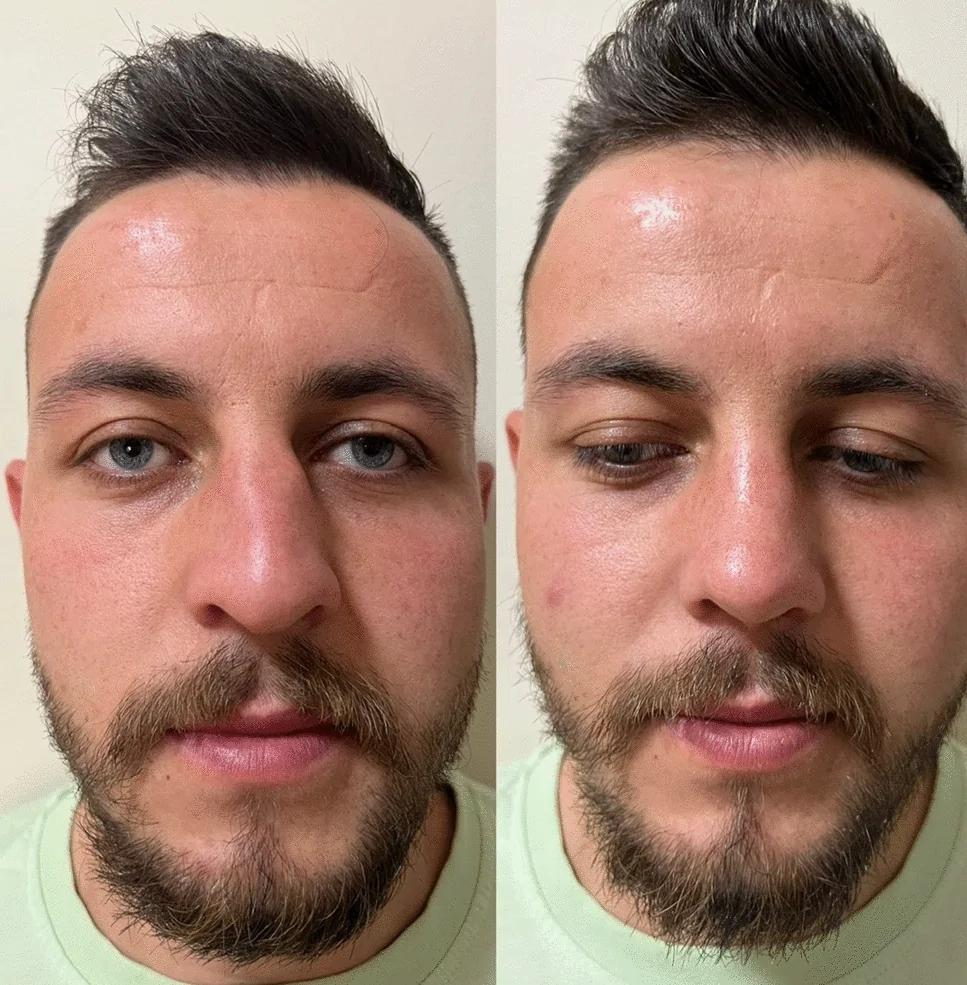
Male patient with reverse C-shaped nasal deviation. Preoperative (left) and postoperative at 24- month follow-up (right) show marked improvement in nasal axis alignment with correction of both dorsal and tip deviation

Patient 4 (Fig. 11): Male patient with C-shaped nasal deviation. Preoperative (left) and postoperative (right) frontal views demonstrate marked improvement in nasal axis alignment. Note the correction of both dorsal and tip deviation, with restoration of midline position at 24-month follow-up.

**Fig. 12:**
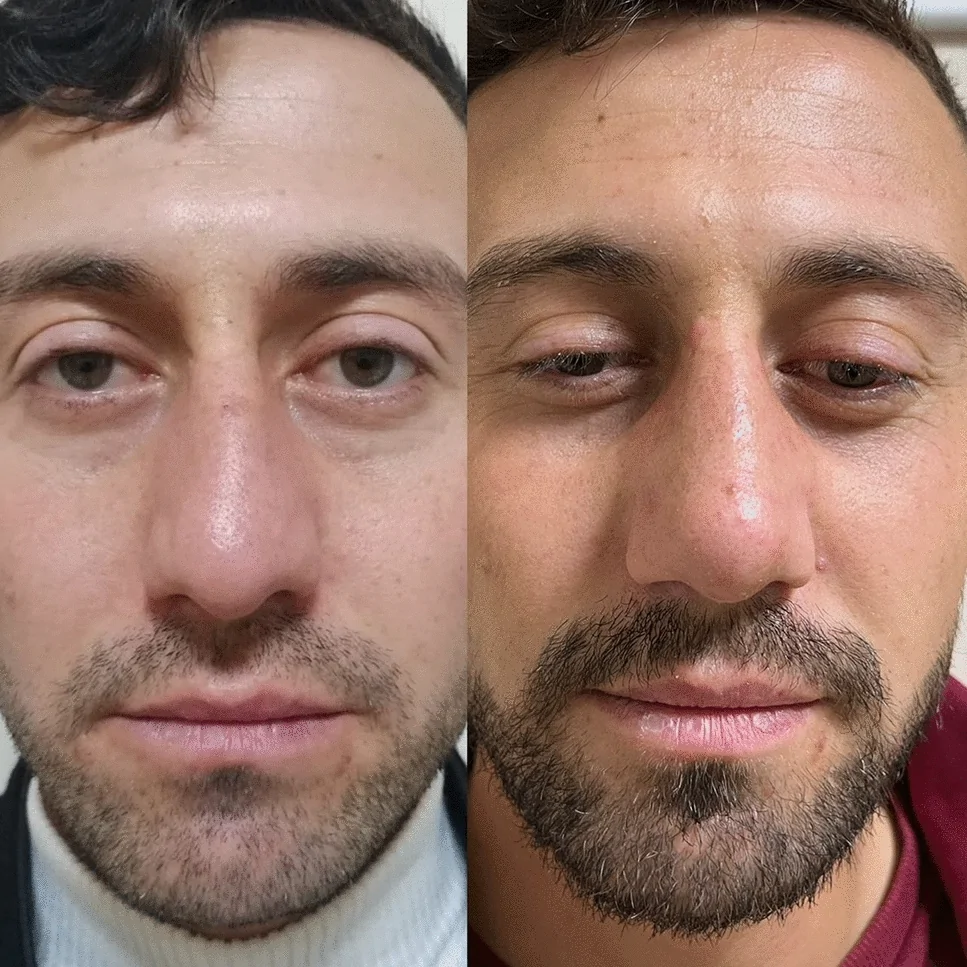
Male patient with reverse C-shaped deviation. Preoperative (left) and postoperative at 18- month follow-up (right) demonstrate effective correction of crooked nose deformity with improved nasal symmetry and aesthetic balance

Patient 5 (Fig. 12): Male patient with reverse C-shaped deviation. Preoperative (left) and postoperative (right) comparison shows effective correction of the crooked nose deformity with improved nasal symmetry and aesthetic balance at 18-month follow-up.

## Discussion

This study demonstrates that the asymmetric puzzle spreader graft technique combined with asymmetric osteotomy provides effective correction of crooked nose deformity, as evidenced by significant improvements in both RDA and NDA measurements. Our results show a 56.7% reduction in rhinion deviation and a 73.7% reduction in nasal tip deviation, with a mean follow-up of 2 years, indicating comprehensive and durable correction throughout the nasal length.

### Rationale for Asymmetric Approach

The fundamental principle underlying our technique is the recognition that crooked nose deformity is inherently asymmetric, involving differential structural abnormalities on the convex versus concave sides [1, 2]. Traditional symmetric spreader graft placement fails to address this asymmetry and may even perpetuate the deformity by applying equal forces to unequal structures [8, 9].

Our approach of placing an extended spreader graft on the wider convex side and a shorter graft on the narrower concave side is based on several biomechanical principles:

- *Differential Structural Support*: The convex side typically exhibits greater collapse and requires more extensive structural support to maintain correction [10]. Our extended graft reaching the radix provides comprehensive support along the entire dorsal length on this side.
- *Asymmetric Force Distribution*: By varying graft length and placement, we create asymmetric counterforces that neutralize the deforming forces responsible for the deviation [15]. The shorter graft on the concave side prevents over-correction while maintaining adequate internal valve support.
- *Preservation of Nasal Valve Function*: The extended graft on the convex side provides robust internal valve support, while the shorter graft on the concave side prevents over-correction and maintains physiologic valve dimensions [7].
- *Interlocking Stability*: The “puzzle piece” configuration, where grafts are carved with complementary shapes to interlock, creates a stable, integrated framework that resists recurrent deviation and provides balanced support across the entire nasal dorsum [11, 12].

### Comparison with Existing Techniques

Our results are consistent with the recent literature emphasizing asymmetric approaches to crooked nose correction. Demir et al. reported similar improvements using asymmetric level osteotomy combined with unilateral spreader graft, achieving RDA reduction from 6.2° to 2.44° (60.6% improvement) and NDA reduction from 5.08° to 2.13° (58.1% improvement) [1]. Our technique differs by employing bilateral but asymmetric grafts rather than unilateral grafting, which we believe provides more balanced structural support and may explain our superior tip correction (73.7% vs. 58.1%).

Moosavizadeh et al. demonstrated that convex-side spreader graft placement produced superior outcomes compared to concave-side placement [10]. Our technique incorporates this principle by placing a larger, extended graft on the convex side while adding a shorter graft on the concave side to provide comprehensive bilateral support and prevent secondary deformities. This bilateral approach may contribute to the durability of correction observed at 2-year follow-up (Table 1).

**Table 1:** Patient demographics and baseline characteristics

Characteristic	Value
Total patients, n	50
Age, years (mean ± SD)	35.4 ± 8.6
Age range, years	18-50
Gender, n (%)	
- Male	27 (54%)
- Female	23 (46%)
Deviation type, n (%)	
- C-shaped	28 (56%)
- Reverse C-shaped	22 (44%)
Mean follow-up, years (range)	2.0 (1-3)
Surgeon	Single surgeon (E.Y.)

The concept of “puzzle grafts” or separated spreader grafts has been described by Uzun et al. [11] and Elsayed et al. [12]. Our technique shares the principle of customized asymmetric grafting but specifically defines the differential graft placement strategy: extended graft to the radix on the convex side versus shorter graft from the K-point on the concave side, with complementary shapes that interlock to create a stable framework (Table 2).

**Table 2:** Comparison of preoperative and postoperative deviation angles

Parameter	Preoperative Mean (Range)	Postoperative Mean (Range)	Mean Improvement	% Reduction	P value*
RDA (degrees)	7.28 (3.5–17.46)	3.15 (1.1–4.2)	4.13	56.7%	<0.05
NDA (degrees)	4.67 (2.5–13.01)	1.23 (0.45–3.21)	3.44	73.7%	<0.05

*RDA* rhinion deviation angle, *NDA* nasal tip deviation angle

^*^Statistical analysis performed using Wilcoxon signed-rank test (IBM SPSS Statistics version 20.0). Data were not normally distributed based on Shapiro–Wilk test

Gökler and Koçak reported successful outcomes combining spreader flap with asymmetric spreader graft [2]. While their technique incorporates spreader flaps, our approach uses cartilaginous grafts exclusively, which may be preferable in cases where mucosal preservation is challenging or where greater structural support is needed (Table 3).

**Table 3:** Functional outcomes—NOSE score comparison

Parameter	Preoperative	Postoperative (1 year)	Mean Improvement	% Reduction	P value*
NOSE Score (mean ± SD)	62.57 ± 19.44	21.84 ± 16.81	40.73	65.1%	<0.001
NOSE Score (median)	71	16	55	77.5%	<0.001

*NOSE* Nasal Obstruction Symptom Evaluation (scale 0-100; higher scores indicate greater obstruction)

^*^Statistical analysis performed using Wilcoxon signed-rank test (IBM SPSS Statistics version 20.0)

Kim et al. demonstrated that one-unit osteotomy produced 70.3% improvement in bony vault deviation [18]. Our 56.7% RDA improvement is comparable to conventional osteotomy outcomes, but our technique excels in tip correction (73.7% NDA improvement), suggesting that the asymmetric puzzle spreader graft configuration is particularly effective for lower nasal deviation (Table 4).

**Table 4:** Complications and adverse events

Complication	Number of Patients	Percentage
Wound infection (minor, resolved with oral antibiotics)	1	2%
Postoperative nasal bleeding (minor, managed conservatively)	2	4%
Revision surgery required	1	2%
Graft displacement	0	0%
Graft extrusion	0	0%
Major complications	0	0%
Total patients with any complication	4	8%

### Role of Osteotomy

The combination of asymmetric osteotomy with asymmetric spreader grafting is crucial for comprehensive correction. Osteotomy addresses the bony component of the deviation by mobilizing the nasal bones and allowing repositioning [16, 17]. The spreader grafts then maintain this corrected position while supporting the cartilaginous framework.

Our asymmetric osteotomy approach customizes the osteotomy pattern to address the specific bony asymmetry present in each case, similar to principles described by Kim et al. [18]. The synergy between asymmetric osteotomy and asymmetric spreader grafting provides correction of both bony and cartilaginous components, which is essential for achieving optimal outcomes in crooked nose deformity.

### Objective Measurement and Validation

The use of objective measurement tools such as RDA and NDA is essential for evidence-based evaluation of surgical outcomes [13, 14]. Unlike subjective assessments, these angle measurements provide reproducible, quantifiable data that allow for meaningful comparison across studies and techniques.

Our use of GIMP software for angle measurement has been validated in previous studies [13]. Alternative methods include AutoCAD, GeoGebra, and three-dimensional imaging systems [14, 19]. Rendón-Medina et al. found that 2D photographs, low-cost 3D surface imaging, and CT are broadly comparable for systematic nasal analysis [20], supporting our use of 2D photographic analysis as a valid and accessible method.

The significant improvements in both RDA and NDA in our study provide objective evidence of the technique’s efficacy. The greater percentage reduction in NDA (73.7%) compared to RDA (56.7%) suggests that the technique is particularly effective in correcting lower nasal deviation, which is often the most aesthetically concerning component of crooked nose deformity. This superior tip correction may be attributed to the bilateral support provided by the asymmetric grafts, which extends structural correction throughout the entire nasal length.

### Biomechanical Considerations

The asymmetric puzzle spreader graft technique addresses several key biomechanical factors in crooked nose deformity:

- *Dorsal Septal Tension:* The extended graft on the convex side helps redistribute dorsal septal tension, counteracting the forces that pull the dorsum off midline [3, 21]. By extending to the radix, the graft provides comprehensive control of dorsal alignment.
- *Lateral Wall Width Asymmetry:* By combining asymmetric osteotomy with differential graft placement, we equalize lateral wall widths and create a more symmetric nasal framework [18].
- *Internal Valve Geometry:* The grafts restore appropriate internal valve angles on both sides while accounting for the pre-existing asymmetry [7, 22]. The bilateral approach ensures that both valves receive adequate support.
- *Structural Integrity:* The interlocking “puzzle” configuration of the grafts creates a stable, integrated framework that resists recurrent deviation [11, 12]. The complementary shapes of the grafts lock together, providing mutual reinforcement and preventing displacement.

### Functional Outcomes and Quality of Life

The substantial improvement in NOSE scores (65.1% reduction, P < 0.001) demonstrates that the asymmetric puzzle spreader graft technique provides significant functional benefit in addition to aesthetic correction. This finding is particularly important as crooked nose deformity is frequently associated with nasal obstruction due to internal valve compromise and septal deviation [7, 22].

The mean postoperative NOSE score of 21.84 indicates that most patients achieved mild or minimal nasal obstruction symptoms following surgery. This outcome compares favorably with published functional rhinoplasty results. Most et al. reported that NOSE scores below 30 generally indicate successful functional outcomes [22]. Our results demonstrate that 65.1% improvement in NOSE scores, which exceeds many published benchmarks for functional rhinoplasty.

The improvement in NOSE scores validates our technique’s dual benefit: The asymmetric spreader grafts not only correct the aesthetic deformity but also restore internal valve function and improve nasal airway patency. The extended graft on the convex side provides robust internal valve support, while the shorter graft on the concave side prevents over-correction that could compromise the airway. This balanced approach ensures optimal functional outcomes alongside aesthetic correction.

### Long-term Durability

Our mean follow-up of 2 years (range: 1-3 years) provides evidence of the durability of correction achieved with this technique. Only one patient (2%) required revision surgery at 18 months, indicating that the asymmetric puzzle spreader graft configuration provides stable, long-term correction. The low revision rate compares favorably with published series and suggests that the interlocking graft configuration effectively resists the forces that commonly lead to recurrent deviation.

### Limitations and Future Directions

*Several limitations of this study should be acknowledged*:

- *Retrospective Design*: The retrospective nature limits our ability to control for all variables and establish definitive causation. Prospective randomized controlled trials comparing asymmetric puzzle spreader grafts versus symmetric spreader grafts, unilateral grafts, and other asymmetric techniques would provide higher-level evidence.
- *No Control Group*: Without a direct comparison group, we cannot definitively establish that our outcomes are specifically attributable to the “puzzle” configuration versus general principles of asymmetric grafting and osteotomy. However, our outcomes compare favorably with published benchmarks.
- *Lack of Functional Outcomes*: While we measured anatomical correction, we did not systematically assess functional outcomes such as nasal airflow or patient-reported breathing improvement using validated instruments such as the NOSE scale or rhinomanometry. Future studies should incorporate these measures to correlate structural correction with functional improvement.
- *Single-Center, Single-Surgeon Experience*: Our results reflect the experience of a single surgical team. Multi-center studies would help validate the generalizability of our findings and assess the reproducibility of the technique across different surgeons.
- *Heterogeneity of Deformities*: While we analyzed C-shaped and reverse C-shaped deformities together, subgroup analysis based on deformity type, severity, and etiology would help identify which patients benefit most from this technique.

*Future research directions include*:

- Prospective comparative studies evaluating asymmetric puzzle spreader grafts versus other asymmetric and symmetric techniques
- Long-term follow-up studies (≥5 years) to assess durability of correction and identify any late complications or recurrence
- Integration of functional outcome measures (NOSE scale, rhinomanometry) and patient-reported outcomes (FACE-Q, ROE)
- Three-dimensional imaging analysis to provide more comprehensive assessment of nasal symmetry
- Finite element modeling to better understand the biomechanical effects of asymmetric graft placement and interlocking configuration
- Development of standardized protocols for determining optimal graft dimensions based on preoperative measurements
- Multi-center validation studies to establish reproducibility across different surgeons and patient populations

### Clinical Implications

*The asymmetric puzzle spreader graft technique offers several clinical advantages*:

- *Tailored Correction*: By customizing graft size and placement to each side’s specific anatomy, the technique addresses the fundamental asymmetry of the deformity in a biomechanically sound manner.
- *Comprehensive Approach*: The combination with asymmetric osteotomy provides correction of both bony and cartilaginous components, addressing the entire pathoanatomy of crooked nose deformity.
- *Objective Validation*: The significant improvements in RDA (56.7%) and NDA (73.7%) provide evidence-based validation of the technique’s efficacy, with particularly strong tip correction.
- *Reproducibility*: The clearly defined principles (extended graft to radix on convex side, shorter graft from K-point on concave side, interlocking “puzzle” configuration) make the technique reproducible across different surgeons.
- *Low Morbidity*: Using autologous septal cartilage grafts minimizes the risk of infection, extrusion, or foreign body reaction. Our low complication rate (2% infection, 4% minor bleeding, 2% revision) supports the safety of this approach.
- *Durability*: Mean 2-year follow-up with only 2% revision rate demonstrates long-term stability of correction.
- *Balanced Support***:** The bilateral approach provides balanced structural support across the entire nasal framework, potentially contributing to the superior tip correction and long-term stability observed in our series.

## Conclusions

The asymmetric puzzle spreader graft technique combined with asymmetric osteotomy and osteoplasty provides effective correction of C-shaped and reverse C-shaped crooked nose deformities with durable results at 2-year follow-up. Our study demonstrates significant improvements in both rhinion deviation angle (56.7% reduction) and nasal tip deviation angle (73.7% reduction), providing objective evidence of the technique’s efficacy with particularly strong tip correction.

By placing an extended spreader graft reaching the radix on the wider convex side and a shorter graft from the K-point on the narrower concave side, configured to interlock like puzzle pieces, this technique addresses the inherent asymmetry of crooked nose deformity in a tailored, biomechanically sound manner. The “puzzle” configuration of interlocking asymmetric grafts creates a stable, integrated framework that effectively maintains correction with low complication and revision rates.

This technique represents a valuable addition to the rhinoplasty surgeon’s armamentarium for managing one of the most challenging deformities in facial plastic surgery. The use of objective measurement tools such as RDA and NDA allows for evidence-based assessment and comparison of outcomes. The bilateral asymmetric approach may provide advantages over unilateral grafting by offering more balanced structural support, potentially explaining the superior tip correction observed in our series.

Further prospective comparative studies with longer follow-up and inclusion of functional outcome measures will help to fully establish the role of this technique in the management of crooked nose deformity and identify which patients benefit most from this approach.

## Data Availability

Available.
